# Different Biomechanical Variables Explain Within-Subjects Versus Between-Subjects Variance in Step Length Asymmetry Post-Stroke

**DOI:** 10.1109/TNSRE.2021.3090324

**Published:** 2021-06-29

**Authors:** Natalia Sánchez, Nicolas Schweighofer, James M. Finley

**Affiliations:** Division of Biokinesiology and Physical Therapy, University of Southern California, Los Angeles, CA 90089 USA; Division of Biokinesiology and Physical Therapy, Department of Biomedical Engineering, University of Southern California, Los Angeles, CA 90089 USA, and also with the Neuroscience Graduate Program, University of Southern California, Los Angeles, CA 90089 USA; Division of Biokinesiology and Physical Therapy, Department of Biomedical Engineering, University of Southern California, Los Angeles, CA 90089 USA, and also with the Neuroscience Graduate Program, University of Southern California, Los Angeles, CA 90089 USA

**Keywords:** Gait, stroke, step length asymmetry, dimensionality reduction, regression

## Abstract

Step length asymmetry (SLA) is common in most stroke survivors. Several studies have shown that factors such as paretic propulsion can explain between-subjects differences in SLA. However, whether the factors that account for between-subjects variance in SLA are consistent with those that account for within-subjects, stride-by-stride variance in SLA has not been determined. SLA direction is heterogeneous, and different impairments likely contribute to differences in SLA direction. Here, we identified common predictors between-subjects that explain within-subjects variance in SLA using sparse partial least squares regression (sPLSR). We determined whether the SLA predictors differ based on SLA direction and whether predictors obtained from within-subjects analyses were the same as those obtained from between-subjects analyses. We found that for participants who walked with longer paretic steps paretic double support time, braking impulse, peak vertical ground reaction force, and peak plantarflexion moment explained 59% of the within-subjects variance in SLA. However the within-subjects variance accounted for by each individual predictor was less than 10%. Peak paretic plantarflexion moment accounted for 4% of the within-subjects variance and 42% of the between-subjects variance in SLA. In participants who walked with shorter paretic steps, paretic and non-paretic braking impulse explained 18% of the within-subjects variance in SLA. Conversely, paretic braking impulse explained 68% of the between-subjects variance in SLA, but the association between SLA and paretic braking impulse was in the opposite direction for within-subjects vs. between-subjects analyses. Thus, the relationships that explain between-subjects variance might not account for within-subjects stride-by-stride variance in SLA.

## I. Introduction

GAIT impairment is common in the majority of survivors of stroke. A common measure of gait impairment post-stroke is step length asymmetry (SLA). SLA is associated with increased cost of transport [1]–[3] and decreased balance [4], [5], making SLA reduction a common goal of clinical interventions [6], [7]. Studies assessing factors associated with SLA post-stroke rely on average measures obtained over multiple strides for each participant, and use techniques such as univariate correlation [8]–[10], analysis of variance [9], [11], [12], or linear regression [8], [13], [14] to understand factors that explain between-subjects differences in SLA. These studies have identified factors related to forward propulsion, such as paretic propulsion [10], [15], [16], plantarflexion moments [9], and trailing limb extension [17], as primary correlates of SLA. However, whether these between-subjects relationships hold at a within-subjects level remains to be determined.

SLA is highly heterogeneous. Some people walk with asymmetries characterized by longer paretic steps, others walk with asymmetries characterized by shorter paretic steps, and other individuals walk with nearly symmetric steps [1], [16]. Asymmetries characterized by longer paretic steps are thought to be related to decreased propulsion and limb extension when the paretic limb is trailing [8]–[11]. In contrast, asymmetries characterized by shorter paretic steps are thought to be related to deficits in paretic limb advancement [1]. However, no study has systematically identified the factors contributing to each type of step length asymmetry.

Laboratory-based gait analysis can return tens of variables, which can be difficult to synthesize to understand the contributors to gait asymmetry. For example, one can quantify spatiotemporal variables such as stance and swing times and step lengths. Additionally, researchers can measure ground reaction forces and joint-level kinematics to obtain joint level kinetics, which are measured continuously throughout the gait cycle [18], [19]. These continuous measures can be used to extract features for each step using peak values, ranges, or impulses. All of these variables are then typically averaged over several gait cycles, eliminating stride-by-stride variance. Researchers will then select a subset of variables to try and explain between-subjects differences in SLA [8], [9], [13]. Therefore, traditional approaches reduce available information when attempting to identify the factors that contribute to SLA by eliminating within-subjects variance using average metrics and reducing the number of variables included in the analyses [20].

Many gait variables are correlated due to the inherent coordination found in the gait pattern. In multivariate linear regression, predictors need to be independent of each other to avoid multicollinearity. Multicollinearity occurs when a predictor is highly correlated with the linear combination of the other predictors. Therefore, the predictor variables’ individual effects on the response cannot be separated [21], making variable selection necessary when defining predictor variables. Variable selection [22] can be based on experimental hypotheses, which, while well-intentioned, can introduce biases because potentially meaningful predictors may be over-looked. Other techniques can reduce researcher bias in variable selection, using criteria such as the variance inflation factor (VIF) [23] or methods that shrink the estimated regression coefficients for a number of variables such as Ridge [24] or Lasso [25]. However, the resulting models obtained from these methods provide no insight into relationships between predictor variables. A combination of dimensionality reduction, to provide insights into relationships between variables, with linear regression to identify SLA predictors might inform on several correlated variables, which could be equally targeted in an intervention aimed to reduce SLA.

Here, we used sparse partial least square regression (sPLSR) [26]–[28], a technique that combines dimensionality reduction and Lasso variable selection, to determine which factors from a set of 20 variables collected for each lower extremity during gait analysis, can predict within-subjects variance in SLA in a sample of individuals with chronic stroke. Our goal was to determine: 1) if the predictors of SLA differ for individuals with asymmetries due to taking shorter paretic steps versus longer paretic steps [1], [8], [29], and 2) if the factors that predict within-subjects variance in SLA are consistent with those that predict group-level variance in SLA [9], [10], [15]–[17]. We hypothesized that for asymmetries characterized by longer paretic steps, SLA would be associated with variables that capture paretic support deficits such as paretic stance time and paretic vertical ground reaction force [1], [16]. For asymmetries characterized by shorter paretic steps, we hypothesized that SLA would be associated with kinematic deficits in paretic limb advancement during swing such as peak hip, knee, or ankle flexion angles [1], [16]. In agreement with previous literature [3], [9], [10], [15]–[17], we hypothesized that measures related to paretic propulsion would predict SLA independent of asymmetry direction. Our results will demonstrate how dimensionality reduction and regression to determine factors that explain within-subjects variance can provide a more complete understanding of within-subjects relationships between SLA and other biomechanical variables to inform novel, individualized intervention targets for rehabilitation of walking after stroke.

## II. Methods

### A. Population

Data used in this study were collected from a convenience sample of individuals post-stroke (Table I) as part of a previous study [1]. We recruited individuals with chronic hemiparetic stroke from the Registry for Aging and Rehabilitation Evaluation (RARE) database at the University of Southern California. Study inclusion criteria were: (1) chronic hemiparesis (time since stroke >6 months) caused by a single stroke, (2) ability to walk on the treadmill continuously for 5 minutes, (3) ability to walk over ground independently or with use of a cane, (4) no concurrent neurological disorders or orthopedic conditions that interfered with their ability to walk, and (5) the ability for them or a guardian to provide informed consent. Exclusion criteria were inability to walk, clinical history of more than one stroke, or any orthopedic or neurological condition that prevented them from walking in the last year. All procedures conformed to the principles set forth in the Declaration of Helsinki and were approved by the University of Southern California’s Institutional Review Board.

### B. Experimental Protocol, Data Acquisition and Processing

All data collection took place at the Locomotor Control Lab at the University of Southern California. After obtaining informed consent, we assessed lower-extremity motor impairment using the lower-extremity portion of the Fugl-Meyer (FM) assessment [30]. We then assessed walking speed using the 6-minute walk test (6MWT). After clinical assessments, participants walked on an instrumented, dual belt treadmill (Fully Instrumented Treadmill, Bertec Corporation, OH) for a familiarization trial, where the speed of both belts was gradually adjusted using the staircase method [31] until participants achieved their comfortable walking speed, which they maintained for 3 minutes. After a break, participants completed a 5-minute walking trial on the treadmill, where we measured all gait variables of interest. During all treadmill trials, participants wore a harness to prevent falls without providing any body weight support. Participants were instructed to lightly touch a handrail placed in front of them to aid balance and prevent drift on the treadmill [1].

**Fig. 1.**
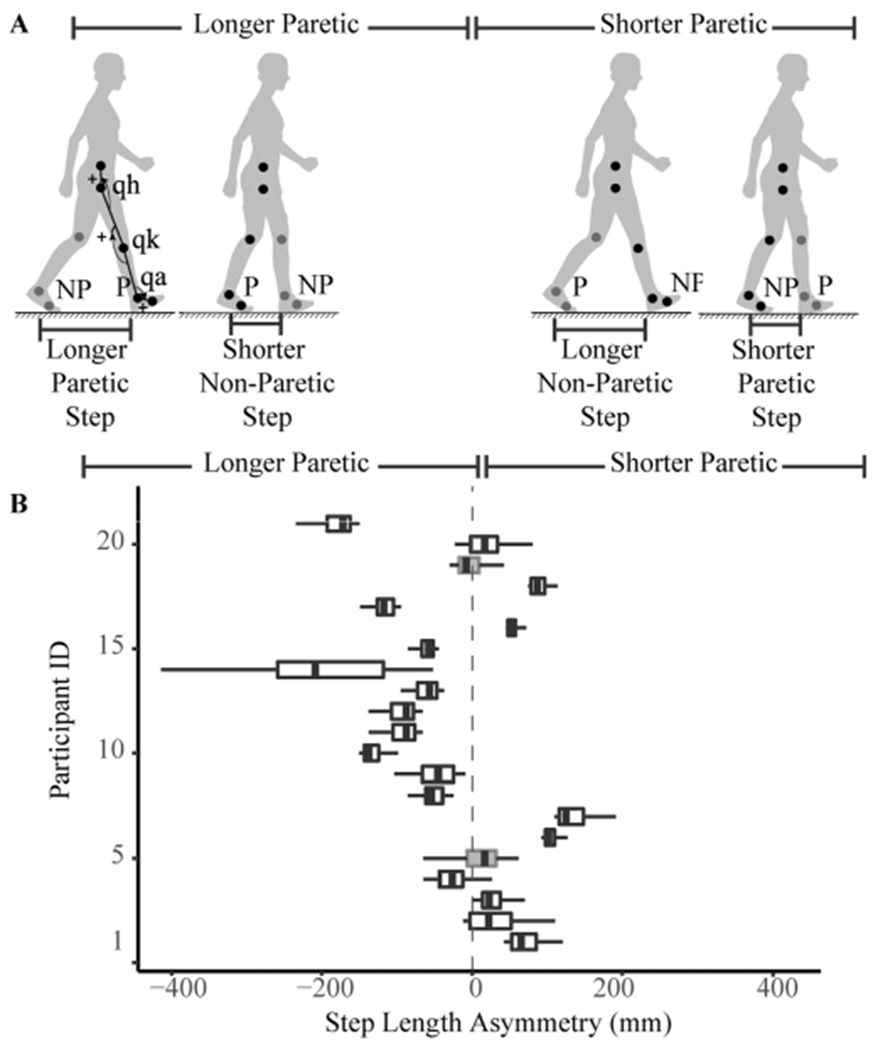
Characterization of SLA. **A)** Participants in our study walked with longer steps with their paretic extremity (left) or with shorter steps with their paretic extremity (right). Location of motion capture markers are indicated in the figure, as well as conventions for measuring joint kinematics (q for ankle, knee and hip). P: paretic. NP: non-paretic Arrowheads indicate direction of positive rotation. **B)** Distribution of SLA observed across participants. Participants whose 95% CI for SLA included zero were excluded from analyses to ease interpretation.

We recorded the position of reflective markers located bilaterally on the metatarsophalangeal joints, lateral malleoli, tibial lateral condyle, greater trochanters, and iliac crests at 100 Hz (Figure 1A) using a 10-camera Qualisys Oqus system (Qualisys AB, Goteborg, Sweden). We recorded ground reaction forces generated by each leg at 1000 Hz from force plates embedded in a dual belt treadmill. Since all participants walked at different speeds, they all took a different number of strides. Thus, we selected 50 strides collected during the five-minute walking trial to provide an equal number of strides for each participant in our analysis. Strides were collected from the mid-portion of the trial by identifying the halfway stride and collecting the 25 strides prior to and after this mid-point.

We used a fourth-order low-pass digital Butterworth filter to smooth marker data using a cutoff frequency of 10 Hz. Step lengths were defined as the fore-aft distance between the lateral malleoli markers at the time of the respective limb’s initial contact [32]–[34]. Initial contact and lift-off were estimated from peak anterior and posterior excursions of the lateral malleoli, respectively [35]. We characterized step length asymmetry as the difference in non-paretic (NP) minus paretic (P) step lengths (SL) in millimeters:

(1) $$SLA=SL_{NP}-SL_{P}$$

For each participant, we obtained the 95% confidence interval of their step length asymmetry. If this interval spanned zero, we excluded the participant from analyses because we would not be able to assign these individuals to either the longer paretic or shorter paretic group. If the 95% CI for SLA was less than zero, participants were categorized as walking with longer paretic steps. Otherwise, participants were categorized as walking with shorter paretic steps. We removed strides in the opposite direction from each participant if needed and then used the magnitude of step length asymmetry (|SLA|) in our subsequent analysis.

To obtain temporal gait variables, we calculated stance, swing and double support times from marker data [35]. Swing time corresponds to the time between toe-off, which was estimated as the most posterior location of the ankle markers, to heel strike on the same side, which was estimated as the most anterior location of the ankle marker. Stance time corresponds to the time between initial contact and foot-off on the same side. Finally, double support time for a given limb corresponds to the time from contralateral initial contact to ipsilateral foot-off. We calculated sagittal plane joint angles using custom code written in MATLAB R2019b (Mathworks, Natick, MA). Joint angles and moments were expressed using the conventions defined in Winter [18]: the foot was defined as the segment between the 5^th^ metatarsophalangeal joint and the lateral malleolus, the shank as the segment between the lateral malleolus and the lateral tibial epicondyle, the thigh as the segment between the lateral tibial epicondyle and the greater trochanter, and the pelvis as the segment between the greater trochanter and iliac crest. Ankle dorsi/plantar flexion angle was measured as the angle between the foot and shank. Knee flexion/extension was defined as the angle between the thigh and shank segments. Finally, the hip angle was defined as the angle between the thigh segment and the pelvis.

We low-pass filtered ground reaction forces at a cutoff frequency of 100 Hz and calculated braking and propulsive impulses for each gait cycle as the area under the curve of the negative and positive portion of the fore-aft ground reaction force, respectively. We estimated flexion/extension joint moments from ground reaction forces and joint kinematics using custom inverse dynamics code written in MATLAB and obtained the magnitude of peak flexion/extension moments for each stride and for each joint.

### C. Statistical Analyses

#### 1) Within-Subjects Analyses:

We used sparse partial least squares regression (sPLSR) [26]–[28] to identify the factors that best predict within and between-subjects variance in |SLA| from the 20 gait variables derived for each lower extremity (40 total, Table II). sPLSR is a technique commonly used in “omics” data [26], [28], [36], and chemometrics [27] and relates one or more response variables with a sparse set of predictors by regressing the response variables on a low-dimensional space derived from the full set of correlated candidate predictors. Similar to sparse principal component analysis (sPCA) [37], sPLSR derives a set of orthogonal latent variables whose elements are a sparse subset of the candidate predictors [26]. Unlike sPCA, which is an unsupervised method, the latent variables in sPLSR maximize the variance explained in a response variable, which we defined as SLA magnitude (|SLA|). Variable selection to obtain the sparse latent variables from the full set of candidate predictors is prescribed using a Lasso approach [25], which shrinks the regression coefficients of some predictors to zero during the singular value decomposition that returns the latent variables. sPLSR can also handle multilevel analysis to identify the predictors that best explain within-subjects variance in |SLA| by including a random intercept term in the model to account for between-subjects differences not captured by the predictor variables, such as walking speed and impairment.

We ran analyses separately in individuals who walked with longer paretic steps and shorter paretic steps. All analyses were run in RStudio with R version 3.6.1. sPLSR analyses used the MixOmics package [36] version 3.11. Data for all participants in each group were combined into two N × 42 matrices with one column consisting of the response variable |SLA|, another column consisting of the participant identifier and the remaining 40 columns consisting of the predictor variables listed in Table II. The number of rows N in each matrix was determined by the number of participants in each group (P) and the number of strides for each participant (S), such that N = P × S. Given that predictor variables include temporal, kinetic, and kinematic variables, which have different magnitudes, we scaled and centered all predictors across participants and expressed them as z-scores. We then used a multilevel sPLSR on the stride-by-stride dataset collected for participants who walked with longer paretic steps and shorter paretic steps separately to identify common fixed-effects that explained within-subjects variance in |SLA|. The response variable |SLA| was not z-scored to allow interpretation of regression coefficients, which should have units of millimeters.

sPLSR requires setting two free parameters: the number of latent variables to define the low-dimensional space and the sparsity, defined as the number of non-zero predictors returned by the Lasso approach in each latent variable. We performed an exhaustive search of every combination of up to 10 latent variables with up to 10 non-zero predictors in each latent variable from the full set of 40 candidate predictors to obtain a low dimensional, sparse model that can be easily interpreted [38]. We used leave-one-out cross-validation (LOOCV) to identify the minimum mean square prediction error (MSPE) for all possible combinations of latent variables and non-zero predictors using custom-written code. We implemented LOOCV, leaving out one observation at a time and setting this observation as the test set while the remaining N − 1 observations constituted the training set. Then, we identified the most sparse model (fewest total predictors) with an MSPE within one standard error of model with the minimum MSPE [21]. We ran this analysis separately for each group. Using the resulting latent variables and predictors in each latent variable from this analysis, we identified the regression coefficients. We identified the variance accounted for (VAF) by each latent variable as the proportion of variance explained by each latent variable divided by the total variance in data.

#### 2) Between-Subjects Analyses:

We obtained averages across all strides for |SLA| and all predictor variables for each participant, as is traditionally done in gait studies. Using the average |SLA|, we split participants into those that walked with longer paretic and those that walked with shorter paretic steps. We z-scored all predictor variables and ran sPLSR analyses for participants who walked with longer paretic and participants who walked with shorter paretic steps separately. We included walking speed as one of the predictors in between-subjects analyses. We set the free parameters using the procedure described in section A but only allowing up to five latent variables with up to five predictors to avoid overfitting the data. We then compared whether the predictors of |SLA| obtained in within-subjects analyses were consistent with those obtained in between-subjects analyses. We assessed model performance as in within-subjects analyses.

#### 3) Validation:

Some of the sPLSR models we identified had a single predictor in each latent variable. This is equivalent to a multivariate linear mixed model. Therefore, we implemented multivariate linear mixed-effects regression to determine whether we obtain the same results as in sPLSR analyses. From the linear model, we can calculate the conditional coefficient of determination (R^2^), which is the variance accounted for by the linear combination of fixed and random effects. We also calculated a modified version of the marginal R^2^ which is the variance explained by the linear combination of fixed effects [39]. The sPLSR package does not return confidence intervals for the regression coefficients. Due to limitations in computational power, we were not able to perform bootstrap analyses of the regression coefficients using sPLSR. Therefore, we derived the 95% confidence intervals for the estimated regression coefficients from the linear mixed-effects models.

Our joint-level metrics were obtained from custom inverse dynamics code. Thus, we validated our results using our custom code to derive inverse dynamics and using data analyzed in Visual3D in nine subjects from a previous study [40]. Visual3D validation details are presented in the Appendix.

## III. Results

The final sample included in our analyses consisted of 19 individuals. Data for two participants were excluded as they walked with both positive and negative SLA, and their mean SLA did not differ from zero (Figure 1B). Eleven participants in our sample walked with SLA characterized by longer paretic steps and shorter non-paretic steps (Figure 1). In these 11 individuals, we accumulated a total of 542 strides, and in this sample, the distribution of |SLA| was right-skewed, with a median of 84 mm and an IQR of 75 mm.

Eight participants in our sample walked with SLA characterized by shorter paretic steps and longer non-paretic steps (Figure 1). In these individuals, we accumulated a total of 371 strides, and |SLA| was normally distributed with an average magnitude of 71 ± 40 mm (mean ± SD).

### A. Predictors of Within-Subjects Variance in SLA

#### 1) *Predictors of* |*SLA*| *for Asymmetries Characterized by Longer Paretic Steps:*

**Fig. 2.**
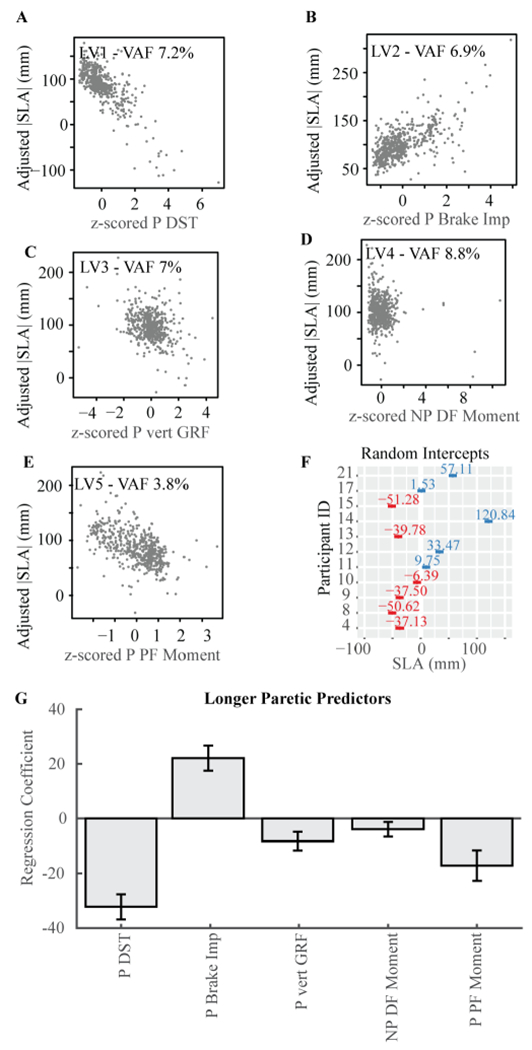
Predictors of |SLA| for participants who walked with longer steps with their paretic leg. **A**-**E)** Conditional regression plots. These plots illustrate the relationship between the expected value for |SLA| when changing the predictor in the x-axis. All other fixed effects are maintained constant at their median value. Note that the figures show combined data for all participants. **F)** Random intercept and 95% CI for each participant. **G)** Estimated regression coefficients for each of the five predictors derived from sPLSR analyses and verified using linear mixed effects model. Error bars are 95% confidence intervals derived from linear mixed effects models described in the Appendix. LV: latent variable. VAF: variance accounted for. DST: double support time.Imp: impulse. Vert: vertical. GRF: ground reaction force. DF: dorsiflexion. PF: plantarflexion.

A model with five latent variables with one predictor each and a random effect term minimized the MSPE of |SLA| for participants who took longer paretic steps (Figure 2). No other models were within one standard error of the model with the minimum MSPE. Using the predictors identified in the sPLSR analyses, we ran a linear mixed model and obtained the same regression coefficients. From the linear mixed model, we calculated the marginal R^2^, which was 0.59. The variance accounted for both the fixed and random effects was 0.84, indicating that 25% of the variance in the model was accounted for by the random intercept. From sPLSR, we obtained the VAF by each individual latent variable composed of a single predictor: (from latent variable 1 to 5, Figure 2A–E, G and Table III) paretic double support time (VAF 7.2%, Figure 2A), paretic braking impulse (VAF 6.9%, Figure 2B), peak paretic vertical ground reaction force (VAF 7%, Figure 2C), peak non-paretic dorsiflexion moment (VAF 8.8%, Figure 2D), and peak paretic plantarflexion moment (VAF 3.8%, Figure 2E). Paretic double support time, peak paretic vertical ground reaction force, peak non-paretic dorsiflexion moment, and peak paretic plantarflexion moment were negatively associated with |SLA| such that larger values for each variable were associated with less asymmetry. Paretic braking impulse was positively associated with |SLA| such that greater braking would be associated with greater asymmetry.

To determine whether the individual-specific differences in |SLA| accounted for by the random effect were due to differences in individual-specific walking speed or impairment measured via the FM score, we used a linear model with the random intercept as the response variable and speed and FM as predictors. The model’s F-statistic was 0.802, p-value = 0.481.

#### 2) Predictors of |SLA| for Asymmetries Characterized by Shorter Paretic Steps:

**Fig. 3.**
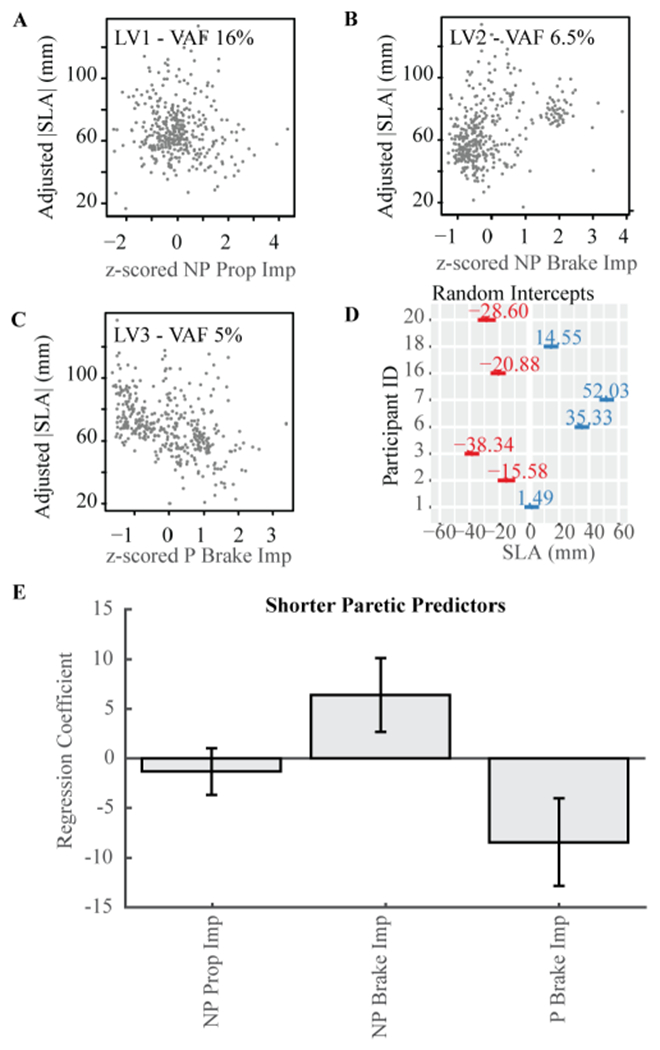
Predictors of |SLA| for participants who walked with shorter steps with their paretic leg. **A**-**C)** Conditional regression plots as in Figure 2. **D)** Random intercept and 95% CI for each participant. **E)** Estimated regression coefficients for each of the three predictors derived from sPLSR analyses and verified using linear mixed effects model. Error bars are 95% confidence intervals. LV: latent variable. VAF: variance accounted for. Prop: propulsion.

A model with three latent variables and one predictor each was within one standard error of the model with the minimum MSPE of |SLA| (Figure 3). The model was also equivalent to a mixed effect model, with a marginal R^2^ of 0.19. The variance accounted for by both the fixed and random effects was 0.77, indicating that 58% of the variance in the model was accounted for by the random intercept. The predictors that made up each latent variable and the VAF derived from sPLSR (from latent variable 1 to 3, Figure 3A–C and Table III) were: non-paretic propulsive impulse (VAF 16%, Figure 3A), non-paretic braking impulse (VAF 6.5%, Figure 3B), and paretic braking impulse (VAF 5%, Figure 3C). Paretic braking impulse had the largest regression coefficient magnitude and was negatively associated with |SLA| (Figure 3C). Non-paretic braking impulse was positively associated with |SLA|. The regression coefficient for non-paretic propulsive impulse was not significantly different from zero.

The linear model to predict the random intercept as a function of speed and FM was not significant, with an F-statistic of 0.141, p-value = 0.871.

### B. Predictors of Between-Subjects Variance in SLA

#### 1) *Predictors of* |*SLA*| *for Asymmetries Characterized by Longer Paretic Steps:*

**Fig. 4.**
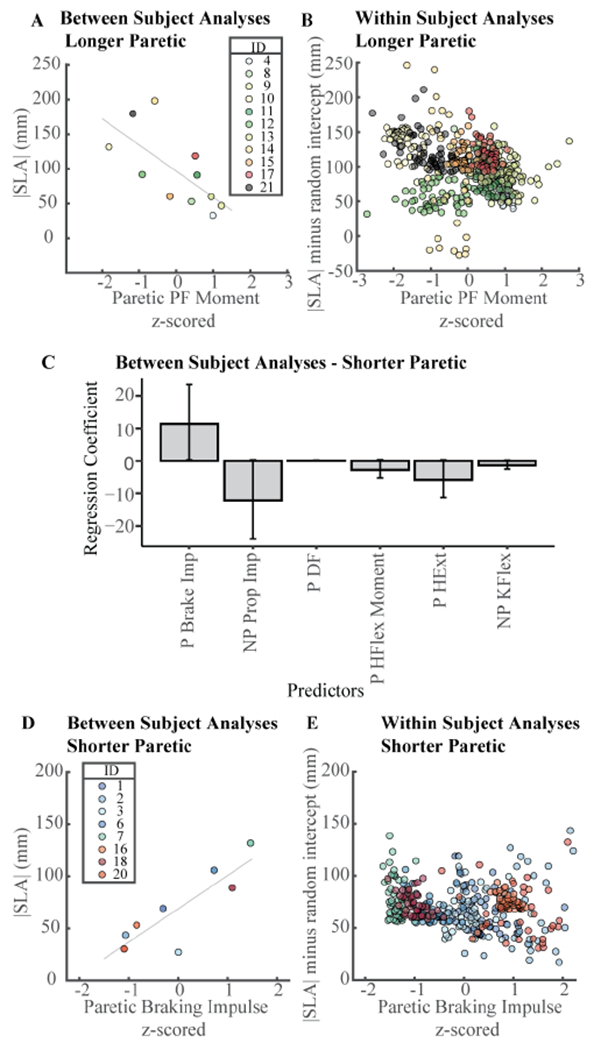
Between-subjects vs. within-subjects relationships between |SLA| and biomechanical variables. **A)** Between-subjects relationship between |SLA| and peak paretic plantarflexion moment in individuals who walked with longer steps with their paretic extremity. **B)** Within-subjects relationship between |SLA| and peak paretic plantarflexion moment in individuals who walked with longer steps with their paretic extremity. |SLA| was adjusted using each individual’s random intercept. **C)** Regression coefficients for the model with two latent variables and three predictors that explains between-subjects variance in |SLA|. **D)** Between-subjects relationship between |SLA| and paretic braking impulse in individuals who walked with longer steps with their paretic extremity. **E)** Within-subjects relationship between |SLA| and paretic braking impulse in individuals who walked with shorter steps with their paretic extremity. |SLA| was adjusted using each individual’s random intercept.

Using a sPLSR model on average data for each participant to explain between-subjects variance in |SLA|, we identified a model with peak paretic plantarflexion moment as a single predictor to be within one standard error of the model with the minimum MSPE. This is equivalent to a univariate linear regression model. When regressing | SLA| onto peak paretic plantarflexion moment, we obtained a model with an intercept of 97(95% CI[71, 123], p = 3.09 × 10^−5^) and slope of −37.8(95% CI[−64.9, −10.7], p = 0.012) (Figure 4A). This indicates that participants with larger average peak paretic plantarflexion moments walked with less average |SLA|. The linear model had an R^2^ of 42%, compared to the VAF for peak paretic plantarflexion in sPLSR of 3.8% when assessing within-subjects variance (Figure 4B).

The sPLSR algorithm did not identify speed as a predictor of |SLA|. These results were verified in a linear model, where the estimated regression coefficient for speed did not differ from zero (p = 0.850).

#### 2) *Predictors of* |*SLA*| *for Asymmetries Characterized by Shorter Paretic Steps:*

A model with two latent variables and three predictors each was within one standard error of the model with the minimum MSPE for predicting between-subjects differences in |SLA| in the eight participants who walked with |SLA| characterized by shorter paretic steps (Figure 4C). Latent variable one was composed of paretic braking impulse, non-paretic propulsive paretic hip flexion moment, and peak non-paretic knee flexion. sPLSR did not identify speed as a predictor of |SLA|.

The sPLSR VAF by all latent variables summed up to 95% and is likely overfitting the data as this analysis is done with eight observations. To calculate the confidence intervals of the sPLSR model parameters, we created 1,0000 new samples by sampling participants with replacement and ran bootstrap analyses. The confidence intervals of all predictors, except for paretic braking, spanned zero, further evidencing that this model was over-fitting the data (Figure 4C).

To determine whether predictors that account for between-subjects variance in |SLA| similarly account for within-subjects variance, we used only paretic braking impulse in univariate regression (Figure 4C). When regressing |SLA| onto paretic braking impulse in a between-subjects analysis, we obtained a model with an adjusted R^2^ of 68% and a slope and intercept of 32 (95% CI[15, 49], p = 0.004) and 68 (95% CI[53, 85], p = 4.27 × 10^−5^), respectively. However, when regressing within-subjects |SLA| onto paretic braking impulse, we obtained a model with a slope and intercept of −9 (95% CI[−14, −5], p = 2.05 × 10^−5^) and 69 (95% CI[50, 88], p = 7.08 × 10^−12^), respectively. Since the slopes of the relationships between |SLA| and paretic braking impulse have opposite signs, this supports our conclusion that between-subjects associations might not hold for within-subjects analysis (Figure 4 C–D).

## IV. Discussion

Step length asymmetry is a common, simple measure of gait impairment post-stroke [41], [42]. Researchers have consistently identified measures related to paretic propulsion [9], [10], [15]–[17] as a primary factor explaining between-subjects differences in |SLA|. Here, we used sPLSR to identify common factors across participants that account for within-subjects variance in |SLA| from a set of 40 variables collected during gait analysis. We found that the factors that account for within-subjects variance in |SLA| depend on the direction of asymmetry. In individuals who walked with asymmetries characterized by longer paretic steps, variance in |SLA| was explained by paretic double support time, paretic braking impulse, peak vertical component of the paretic ground reaction force, peak paretic plantarflexion moment, and peak non-paretic dorsiflexion moment. In participants who walked with asymmetries characterized by shorter paretic steps, the resultant predictors of |SLA| were paretic and non-paretic braking impulses. Therefore, the direction of SLA is a factor to consider in the design of rehabilitation interventions aimed at reducing interlimb asymmetry, given the influence of differing biomechanical impairments across asymmetry directions.

Traditionally, researchers will average individual strides to identify between-subjects associations among biomechanical variables. We wanted to determine whether these between-subjects relationships also hold within-subjects. Using sPLSR, we identified peak paretic plantarflexion moment as the single predictor of between-subjects variance in |SLA| for participants who walked with longer paretic steps. Peak paretic plantarflexion moment had an R^2^ = 42%, comparable to previous studies that reported r = −0.785 [10]. In contrast, peak paretic plantarflexion moment only accounted for ~4% of the common within-subjects variance. This low variance accounted for indicates that the relationship between plantarflexion and SLA does not hold at a within-subjects for all participants. Similarly, in participants who walked with shorter paretic steps, between-subjects analyses showed that paretic braking impulse was positively associated with |SLA| with and R^2^ = 68%. In contrast, in within-subjects analyses, paretic braking impulse was negatively associated with |SLA| and accounted for 5% of the within-subjects variance. Therefore, group level, between-subjects relationships between |SLA| and biomechanical variables are not consistently observed at an individual, within-subjects level. These results support the idea that individual characterization of within-subjects variance might aid identify targets for walking interventions post-stroke.

We hypothesized that |SLA| would be negatively associated with paretic support and propulsion in people with |SLA| characterized by longer paretic steps. Our results partially support our experimental hypotheses. Specifically, the third latent variable was composed of peak vertical ground reaction force on the paretic extremity, a proxy for paretic support, and accounted for 7% of the common within-subjects variance in |SLA| with a negative association with |SLA|. This is consistent with the idea that participants take a shorter non-paretic step due to decreased loading capacity during paretic stance. These results contrast previous studies that did not observe a between-subjects correlation between |SLA| and vertical ground reaction force asymmetry [29]. Note that in this previous study, the authors used a force asymmetry index, which is the ratio of paretic to non-paretic vertical ground reaction force. A plausible explanation for this discrepancy might be that the relationship between |SLA| and paretic support holds on a within-subjects level but not between-subjects, or that using an asymmetry ratio eliminates some of the common variance between |SLA| and the paretic ground reaction force.

We hypothesized that |SLA| would be negatively associated with paretic propulsion similar to what has been reported in the literature [9], [10], [15], [16]. However, our results indicate that paretic plantarflexion moment accounted for only ~4% of the within-subjects variance in |SLA|. The question is then, why have previous studies that targeted paretic propulsion using fast walking and functional electrical stimulation (FastFES) effectively reduced step length asymmetry post-stroke [3], [43]? It might be the case that targeting paretic propulsion is an effective strategy to reduce SLA in some individuals, while in others, it might lead to secondary effects in other variables that influence |SLA|, such as those identified here. It is also worth noting in the FastFES study, only 28/42 individuals reduced |SLA| after FastFES [3]. Analyzing within-subjects variance for an intervention of this type could potentially help researchers identify individuals who might respond most favorably to this type of treatment.

In participants who walked with longer paretic steps, we identified paretic braking impulse as a predictor of |SLA|, with a positive association between braking impulse and |SLA|. This relationship is consistent with our understanding of gait mechanics: bringing the paretic leg further forward results in a longer paretic step and an increase in the posteriorly directed component of the ground reaction force [44], [45]. Braking can also be modulated by changing the center of pressure: if the paretic loading and orientation of the paretic limb is constant, but initial contact is achieved with the forefoot, the fore-aft component of the ground reaction force, and thus, braking would increase [46]. It is evident how stroke might lead to increased braking: decreased paretic dorsiflexion leads to initial contact occurring with the fore-foot or with a flat foot. Modulating how initial contact is achieved could contribute to reducing paretic braking without decreasing paretic step lengths. Excessive braking might also imply that in some post-stroke participants, gait is terminated at each paretic step [47] and might need to be restarted with each non-paretic step. Reducing paretic braking would allow non-paretic propulsion to be used not for gait initiation on each step, but to increase forward progression of the non-paretic limb during swing, further reducing step length asymmetry.

In participants who walked with longer paretic steps, paretic double support time comprised the first latent variable and was negatively associated with |SLA|. Here, we defined paretic double support as the period when the paretic extremity is trailing [48]. In people post-stroke, double support time is longer on the paretic extremity because non-paretic heel strike occurs earlier in the gait cycle [48]. Based on the negative association between double support time and |SLA|, an increase in double support time would lead to reductions in |SLA|. Our interpretation of this association is that increased double support time on the paretic extremity would result in an increase in paretic trailing limb angle. Thus, on the next paretic swing phase, since push-off occurred further behind the body, if the excursion of the paretic leg is constant, the paretic leg would land closer to the body, decreasing paretic step length and subsequently reducing asymmetry.

Finally, in participants who walked with longer paretic steps, a common predictor of within-subjects variance in |SLA| was non-paretic dorsiflexion. Peak non-paretic dorsiflexor moment was negatively associated with |SLA|, and the asymmetry in this group is not only due to a longer paretic step but also a shorter non-paretic step. Thus, a short non-paretic step results in an initial contact closer to the body such that the tibialis is less stretched and cannot generate the eccentric contraction that produces the dorsiflexion moment during loading response. The muscle action of the pretibialis muscles contributes to shock absorption and the heel rocker responsible for limb progression [19]. Non-paretic dorsiflexion might not serve as a direct rehabilitation target, but might instead be a mechanical consequence of SLA.

In people with asymmetries characterized by shorter paretic steps, we hypothesized that |SLA| would be associated with deficits in paretic limb advancement such as paretic ankle, knee, and hip flexion. Our results contrast our hypothesis as we identified paretic and non-paretic braking impulse as the main predictors of |SLA|. Few studies have assessed the role of braking during locomotion in people post-stroke [45], and associations between |SLA| and paretic braking have not been reported in the literature to the best of our knowledge. Here, for participants who walked with shorter paretic steps, paretic braking impulse was negatively associated with |SLA|. Since shorter paretic steps are associated with increased |SLA| in this group, we would expect paretic braking impulse to increase as they take longer paretic steps to reduce |SLA|. In contrast, non-paretic braking was positively associated with |SLA|. Thus, a potential approach for reducing |SLA| could be to reduce non-paretic braking using strategies such as biofeedback of trunk advancement over the non-paretic leg [45], previously shown to be associated with braking.

Overall, each predictor in the sPLSR models accounted for less than 10% of |SLA| variance. There are multiple reasons for the low variance explained by individual predictors. First, sPLSR identifies common predictors that explain within-subjects variance in SLA, but the model cannot account for between-individual differences in the associations between SLA and the candidate predictors. A careful review of Fig. 3 highlights this point: for example, participant 14 shows little variance in peak paretic plantarflexion moment while spanning the entire range of |SLA| values, whereas participant 21 shows a negative association between peak paretic plantarflexion moment and |SLA|. This indicates that the relationships between SLA and biomechanical variables may differ in a subject specific manner. To quantify subject-specific relationships between variables, we would require a model with different predictors for each participant. A final reason why our models accounted for less variability than previous studies [3], [9], [10], [15]–[17], is that these studies use individual averages which remove the noise present in the within-subject data, resulting in between subjects analyses with a higher variance explained. Our results suggest that there are individual-specific correlates of SLA that are not accounted for in between subject analyses, or even in within-subject analyses that combine hierarchical, dimensionality-reduction and regression methods as implemented here.

In participants who walked with longer paretic steps, the marginal R^2^ was 59%, whereas in participants who walked with shorter paretic steps the marginal R^2^ was only 19%. This indicates that in individuals who walk with shorter paretic steps, there are additional within-subjects differences not accounted for by the biomechanical variables included here, and could be related to impairments in the underlying neuromuscular control, such as muscle weakness or co-contraction. This might explain why in our previous study, individuals who took shorter paretic steps had decreased capacity to reduce asymmetry [1]. Further biomechanical assessment of these participants could aid in the identification of targets that are specific to people who walk with shorter paretic steps.

We explored whether differences in the factors that explained within subject variance in |SLA| were due to inter-individual differences in walking speed and impairment. We found no relationship between the random intercept and walking speed or FM, indicating that |SLA| differences between participants were not due to individual differences in walking speed or impairment measured using the FM score. In the between-subjects analyses, speed was not identified as a predictor either by the sPLSR algorithm or during validation via linear models. Therefore, while walking speed and the degree of impairment can influence the magnitude of the biomechanical variables used as predictors of SLA, we found no association between |SLA| and walking speed or impairment. Whether the predictors of |SLA| would differ when grouping individuals based on walking speed remains to be determined.

There are a number of additional considerations that could guide our future work. First, we used peak values over the entire gait cycle as the primary features of our joint kinematic and kinetic data. Previous studies have subdivided the gait cycle into distinct functional phases [19] and then obtained peak values in these phases [9], [11]. The peak values obtained here might have occurred at any point during the gait cycle and might not occur during the gait phases where specific kinetics and kinematics are functionally needed to accomplish the objectives of each phase of the gait cycle. Thus, the relationship between |SLA| and peak values during functional phases remains to be investigated. In some participants such as participant 15, 16 and 18, there was little variance in SLA, hindering identification of predictors of SLA. Future work could include conditions in which individuals modify their SLA or walk at different speeds to increase variance in the predictor and response variables. Finally, we did not include EMG measures as part of data acquisition but this would be important to consider in future studies interested in muscle-level contributions to gait deviations post-stroke.

## V. Conclusion

Using combined dimensionality reduction, sparsity and regression, we found that the factors that account for within-subjects variance in |SLA| are not consistent with those that account for between-subjects variance in SLA and these predictors depend on the direction of asymmetry. Overall, these results point to the need for developing approaches that take advantage of within-subjects variance, to identify personalized intervention targets for gait retraining.

## Figures and Tables

**TABLE I:** Demographic Information

ID	Sex	Age (yrs)	Ethnicity	Months Post-Stroke	Paretic Side	FM	6MWT Speed (m/s)	TM Speed (m/s)	Average SLA (mm)	Mass (kg)	Stroke Type
*1*^b^	M	58	White	83	Left	20	0.76*	0.48	65	94	Hem
*2*^b^	M	49	Asian	43	Right	25	0.95*	0.55	22	86	Isch
*3*	M	73	Asian	143	Left	25	0.8*	0.6	22	67	Isch
*4*	F	64	White	369	Right	18	0.82	0.6	16	60	Isch
*5*^b^	M	45	Asian	53	Left	25	0.93	0.7	−27	96	Isch
*6*	M	69	White	89	Left	23	0.37	0.37	102	87	Isch
*7*	M	54	White	41	Right	24	0.78	0.65	125	95	Hem
*8*^b^	F	39	Hisp/Lat	324	Right	24	0.82*	0.5	−54	64	Isch
*9*	M	58	White	43	Right	23	0.65	0.4	−45	107	Hem
*10*	F	56	White	85	Right	27	0.58	0.58	−137	58	Hem
*11*	M	63	African American	52	Left	23	0.92	0.65	−87	102	Isch
*12*^b^	F	72	White	154	Right	10	0.49	0.32	−87	47	Hem
*13*	M	67	Hisp/Lat	116	Right	26	0.98	0.8	−56	85	Hem
*14*	M	61	Hisp/Lat	34	Left	16	0.27	0.25	−208	108	Isch
*15*	M	60	Hisp/Lat	169	Right	22	0.6	0.45	−57	92	Hem
*16*	M	33	Hisp/Lat	37	Right	28	0.95	0.95	52	125	Isch
*17*	M	61	African American	52	Right	32	1.0	0.85	−117	76	Isch
*18*^b^	M	56	White	88	Right	19	0.42	0.38	86	105	Hem
*19*	M	62	African American	96	Left	18	0.48	0.35	8	122	Hem
*20*^b^	F	28	Hisp/Lat	25	Left	19	0.17	0.13	16	67	Hem
*21*	M	55	White	15	Left	27	0.85	0.65	−172	80	Isch
***Avg***	**16M**	**57**		**98**	**8 Left**	**22**	**0.71**	**0.53**		**87**	**10 Hem**

b Participant wore an ankle brace during the experiment. Hisp/Lat: Hispanic or Latino. FM: Fugl-Meyer score. 6MWT: six minute walk test. TM: Treadmill. Isch: Ischemic stroke. Hem: Hemorrhagic stroke.

* Participants 1-3 and 8 performed the 10-meter walk test due to space constraints.

**TABLE II:** Predictor Variables

Domain	Variable	Units
*Temporal*	Stance	s
	Swing	
	Double support	
	Peak ankle dorsiflexion	
	Peak ankle plantarflexion	
*Kinematic*	Peak knee flexion	Degrees
	Peak knee extension	
	Peak hip flexion	
	Peak hip extension	
	Peak braking force	
	Peak propulsive force	N/kg
*Ground Kinetics*	Peak vertical ground reaction force	
	Braking impulse	N*s/kg
	Propulsive impulse	
	Peak ankle dorsiflexion	
	Peak ankle plantarflexion	
*Joint Moments*	Peak knee flexion	N*m/kg
	Peak knee extension	
	Peak hip flexion	
	Peak hip extension	

The variables above were used in the sPLSR model as predictor variables. Values were measured for the paretic and non-paretic extremities. Units are shown for reference.

**TABLE III:** Fixed Effects Estimated Coefficients

	Estimate	95% CI	P-Value
***Longer Paretic Steps***			
Intercept	97.218	[66.82, 127.62]	6.95×10^−10^
Paretic double support time	−32.268	[−36.85, −27.69]	1.87×10^−37^
Vertical ground reaction force	−8.277	[−11.65, −4.90]	1.85×10^−6^
Paretic braking impulse	22.072	[17.46, 26.69]	1.65×10^−19^
Non-paretic dorsiflexion moment	−3.989	[−6.64, −1.33]	0.003
Paretic plantarflexion moment	−17.182	[−22.77, −11.59]	2.91×10^−9^
***Shorter Paretic Steps***			
Intercept	69.469	[47.683, 91.081]	2.23×10^−10^
Paretic braking impulse	−8.429	[−13.272, 3.233]	1.19×10^−5^
Non-paretic braking impulse	6.395	[3.9601, 10.682]	0.0008
Non-paretic propulsive impulse	−1.338	[−3.68, 1.106]	0.263

Fixed effects in the mixed effect model used to predict within-subject |SLA|.
